# Effects of Repetitive Transcranial Magnetic Stimulation on Cerebellar Metabolism in Patients With Spinocerebellar Ataxia Type 3

**DOI:** 10.3389/fnagi.2022.827993

**Published:** 2022-04-25

**Authors:** Xin-Yuan Chen, Yan-Hua Lian, Xia-Hua Liu, Arif Sikandar, Meng-Cheng Li, Hao-Ling Xu, Jian-Ping Hu, Qun-Lin Chen, Shi-Rui Gan

**Affiliations:** ^1^Department of Rehabilitation Medicine, The First Affiliated Hospital of Fujian Medical University, Fuzhou, China; ^2^The School of Health, Fujian Medical University, Fuzhou, China; ^3^Department of Neurology, The First Affiliated Hospital of Fujian Medical University, Fuzhou, China; ^4^Department of Radiology, The First Affiliated Hospital of Fujian Medical University, Fuzhou, China; ^5^Department of Neurology, The 900th Hospital of Joint Logistics Support Force of PLA, Fuzhou, China

**Keywords:** cerebellar metabolism, magnetic resonance spectroscopy, repetitive transcranial magnetic stimulation, spinocerebellar ataxia type 3, international cooperative ataxia rating scale

## Abstract

**Background:**

Spinocerebellar ataxia type 3 (SCA3) is the most common autosomal dominant hereditary ataxia, and, thus far, effective treatment remains low. Repetitive transcranial magnetic stimulation (rTMS) can improve the symptoms of spinal cerebellar ataxia, but the mechanism is unclear; in addition, whether any improvement in the symptoms is related to cerebellar metabolism has not yet been investigated. Therefore, the purpose of this study was to investigate the effects of low-frequency rTMS on local cerebellar metabolism in patients with SCA3 and the relationship between the improvement in the symptoms and cerebellar metabolism.

**Methods:**

A double-blind, prospective, randomized, sham-controlled trial was carried out among 18 SCA3 patients. The participants were randomly assigned to the real stimulation group (n = 9) or sham stimulation group (*n* = 9). Each participant in both the groups underwent 30 min of 1 Hz rTMS stimulation (a total of 900 pulses), differing only in terms of stimulator placement, for 15 consecutive days. To separately compare pre- and post-stimulation data (magnetic resonance spectroscopy (MRS) data and the International Cooperative Ataxia Rating Scale (ICARS) score) in the real and sham groups, paired-sample *t*-tests and Wilcoxon’s signed-rank tests were used in the analyses. The differences in the ICARS and MRS data between the two groups were analyzed with independent *t*-tests and covariance. To explore the association between the changes in the concentration of cerebellar metabolism and ICARS, we applied Pearson’s correlation analysis.

**Results:**

After 15 days of treatment, the ICARS scores significantly decreased in both the groups, while the decrease was more significant in the real stimulation group compared to the sham stimulation group (*p* < 0.001). The analysis of covariance further confirmed that the total ICARS scores decreased more dramatically in the real stimulation group after treatment compared to the sham stimulation group (*F* = 31.239, *p* < 0.001). The values of NAA/Cr and Cho/Cr in the cerebellar vermis, bilateral dentate nucleus, and bilateral cerebellar hemisphere increased significantly in the real stimulation group (*p* < 0.05), but no significant differences were found in the sham stimulation group (*p* > 0.05). The analysis of covariance also confirmed the greater change in the real stimulation group. This study also demonstrated that there was a negative correlation between NAA/Cr in the right cerebellar hemisphere and ICARS in the real stimulation group (r = − 0.831, *p* = 0.02).

**Conclusion:**

The treatment with rTMS over the cerebellum was found to induce changes in the cerebellar local metabolism and microenvironment in the SCA3 patients. The alterations may contribute to the improvement of the symptoms of ataxia in SCA3 patients.

## Introduction

Spinocerebellar ataxia type 3 (SCA3), also known as Machado–Joseph disease (MJD), is one of the most common types of autosomal dominant neurodegenerative disorders. The manifestation of SCA3 mainly includes gait ataxia, postural imbalance, dysarthria, dysphagia, diplopia, and peripheral neuropathy. Since the pathogenesis of SCA3 has not been fully illustrated, there has not been an effective treatment for this disease so far (Li et al., 2015; Matos et al., 2019; Costa, 2020). There is a growing number of studies applying repetitive transcranial magnetic stimulation (rTMS) in the treatment of spinocerebellar ataxias (SCAs) that has shown promising results (Farzan et al., 2013; Jhunjhunwala et al., 2013; Dang et al., 2019; Manor et al., 2019; Benussi et al., 2020; França et al., 2020). Researchers have observed a marked improvement in the 10-m walk tests and the number of steps in tandem gait and diplopia, particularly after the rTMS treatment, accompanied by an improvement in limb ataxia, as evaluated using the International Cooperative Ataxia Rating Scale (ICARS) (Benussi et al., 2020). Though rTMS can improve the symptoms of SCAs, the mechanism for the improvement is unclear. The previous studies have shown that the therapeutic effect of rTMS on cerebellar ataxia may be due to either the cerebellar modulation of motor cortex excitability by rTMS involving the cerebellar-thalamus-cortical (CTC) pathway (Sanna et al., 2021) or the reduction of oxidative stress, the increase of cerebellar hemispheric blood flow (Ihara et al., 2005), and the effects on cerebellar-cortical plasticity (Song et al., 2020). The magnetic resonance spectroscopy (MRS) provides a non-ionizing and non-invasive method to measure the alteration of cerebellar metabolism (Hall et al., 2012), which has been widely used in the cross-sectional studies of SCAs and admitted as a reliable means in the assessment of the efficacy of SCAs (Lei et al., 2011; Lirng et al., 2012; Adanyeguh et al., 2015; Krahe et al., 2020). Therefore, applying MRS in the detection of the alteration of cerebellar metabolism before and after the rTMS in the SCA3 patients is suitable.

In view of the foregoing, a prospective, randomized, double-blind, sham-controlled study was conducted to investigate the effects of low-frequency rTMS on local intracerebral metabolism in the patients with SCA3 as well as the possible correlation between the alteration of cerebellar metabolism and the improvement of ataxia.

## Materials and Methods

### Ethical Approval and Patient Recruitment

This study was approved by the ethics committee of the First Affiliated Hospital of Fujian Medical University (MRCTA, ECFAH of [2018]201). The registration was recorded in the Chinese Clinical Trial Registry with a unique identifier: ChiCTR1800020133. All the participants signed the informed consent form and any relevant documents. The recruitment of these participants began in December 2018 and ended in October 2019 at the First Affiliated Hospital of Fujian Medical University. The eligibility criteria for the participants were as follows: (1) patients diagnosed with SCA3 and having detectable clinical manifestations; (2) SCA3 patients aged 20–80 years. The participant exclusion criteria were as follows: (1) diagnosed with concomitant epilepsy and dementia (MMSE < 25) or any unstable medical disorder; (2) undergoing neuroleptics or any other current clinical study; (3) a history of seizure, heat convulsion, head injury, neurosurgical interventions, or any metal in the head (outside the mouth); (4) a history of unstable hypertension; (5) a known history of any metallic particles in the eye, implanted cardiac pacemaker, implanted neurostimulators, surgical clips (above the shoulder line), or medical pumps; (6) a history of frequent or severe headaches; (7) a history of migraine, hearing loss, cochlear implants, drug abuse, or alcoholism; and (8) pregnancy or the possibility of pregnancy. The age of onset was defined as the age at which the symptoms associated with SCA3 were first noted by the patient or a close care provider. The duration of the disease was considered to be the time between the age of onset and the age of initial diagnosis.

### Study Design and Treatment Protocol

This study was a double-blind, prospective, randomized, sham-controlled trial. A total of 18 SCA3 patients were randomly allocated to the real or sham stimulation groups using the random number table method (Lim and In, 2019).

A commercially available stimulator (YIDUIDE CCY-I magnetic field stimulator) was utilized for the stimulation in both groups. This trial was completed by 18 (100%) patients. We allocated nine participants (50%) to the real stimulation group; each participant underwent 30 min of 1 Hz rTMS stimulation (a total of 900 pulses) for 15 consecutive days. A total of 9 participants (50%) were assigned to the sham stimulation group; the parameters in the rTMS prescription were the same as those in the real stimulation group. The stimulation coil was placed tangentially above the scalp and centered on the inion in the real stimulation group or vertically in the sham stimulation group (4 cm to the right of the inion and 4 cm to the left of the inion) (Shiga et al., 2002).

### Evaluation

The evaluation was done by the trained study staff, who were blind to both intervention arms. The primary outcome measurement was the score of the ICARS (Trouillas et al., 1997). The ICARS is a 100-point scale including 19 items divided into four subscales: postural and gait (PG), limb kinetic function (KF), speech disorders (DS), and oculomotor disorders (OMS). The higher the score is, the poorer the performance is considered to be. The participants’ performance was evaluated using the ICARS both before and after the stimulus was applied. The secondary outcome measure was taken on the local cerebellar metabolites consisting of the value of *N*-acetyl aspartate (NAA)/creatine (Cr), and choline complex (Cho)/Cr, detected by a proton magnetic resonance wave (^1^H-MRS), using a 3.0-Tesla Siemens Skyra scanner before and after the stimulation. The routine sagittal T1-weighted, coronal T1-weighted, and axial T2-weighted fluid-attenuated inversion recovery (T2-FLAIR) sequences were adopted in the multi-planar anatomical positioning of the voxel of interest (VOI). The multi-voxel ^1^H-MRS sequence was acquired using the following scan parameters: repetition time (TR) = 1,700 ms, echo time (TE) = 135 ms, bandwidth = 1,200 Hz, voxel size = 6.3 mm × 6.3 mm × 15 mm, and total acquisition time = 6 min 53 s. The VOI for each participant was placed at the largest level of the cerebellum, including the bilateral dentate nucleus, cerebellar hemispheres, and the vermis of the cerebellum. The peak areas for NAA at 2.02 parts per million (ppm), Cho at 3.22 ppm, and Cr at 3.03 ppm were automatically calculated by the post-processing software provided by the machine manufacturer. Then, the metabolite intensity ratios, including NAA/Cr and Cho/Cr, for each voxel were acquired. The selection criteria for the voxels in the VOI were as follows: only the voxel with the largest NAA amplitude and good wave quality was selected for analysis when there were multiple voxels simultaneously excited within the VOI.

### Data Processing and Statistical Analyses

The analysis of the primary outcome was based on the intention-to-treat principle. Due to the lack of MRS data, the secondary outcome was based on as-treated analysis (Langezaal et al., 2021). Data normality was determined by the Shapiro–Wilk tests in all the analyses. For comparisons of the baseline data between the two groups, a Fisher’s exact test was used to analyze gender distribution. To separately compare the pre- and post-stimulation data (MRS data and ICARS score) in the real and sham groups, paired-sample *t*-tests and Wilcoxon signed-rank tests were used in the analyses of normal and abnormal data, respectively. The consecutive variables were analyzed by independent samples *t*-tests for normal data, and Mann–Whitney U tests were used for assessing abnormal data. To confirm the robustness of the primary findings, we used the analysis of covariance to fit additional models with the mean change in the assessment metrics (ICARS and MRS) after the treatment session as the dependent variable, the treatment group as the independent variable, and the corresponding baseline scores as covariates. Pearson correlation analysis was performed to assess the relationship between the MRS data and ICARS scores. The above statistics were analyzed and processed using SPSS 27.0 (SPSS Inc., Chicago, IL, United States). The level of significance was established as *p* < 0.05.

## Results

**FIGURE 1:**
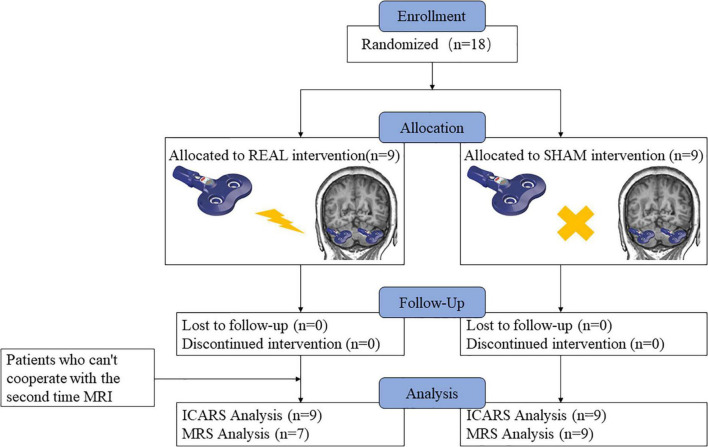
Schematic diagram of the research process. Nine individuals were randomly assigned to the low-frequency rTMS intervention, nine were assigned to the sham intervention, and zero dropped out of the experiment, but two participants in the real stimulation group were not included in the imaging data because they were unable to cooperate with the second MRS examination.

A total of 18 patients participated in this study; nine were randomly assigned to the real stimulation group and nine to the sham stimulation group. No participant dropped out of the study in the sham stimulation group; two participants in the real stimulation group were not included in the imaging data because they were unable to cooperate with the second MRS examination. The flow chart representing all the participants is presented in Figure 1. All the participants tolerated the intervention without significant adverse effects throughout the study. There were no significant differences between the two groups for any of the baseline indicators, including age (*p* = 0.37), gender distribution (*p* = 1.00), age of onset (*p* = 0.38), disease duration (*p* = 0.95), NAA/Cr, Cho/Cr ratios, as well as the ICARS scores (Tables 1–3).

**TABLE 1:** The analysis of baseline data from the patients with SCA3.

Characteristic	Real stimulation group	Sham stimulation group	*P* Value
Number	9	9	−
Age, years	37.78 ± 9.28	41.78 ± 9.18	0.37
Gender, M/F	4/5	4/5	1.00
Age at onset (years)	31.44 ± 9.70	35.56 ± 9.51	0.38
Duration of disease (years)	6.33 ± 3.39	6.22 ± 4.15	0.95

**TABLE 2:** The comparison of scores of ICARS and its four subscales’ domain before and after stimulation in the real and sham intervention groups.

	Real rTMS		Sham rTMS		*P* value*^a^*	*P* value*^b^*	*P* value*^c^*	*P* value*^d^*
				
Variables	Pre-rTMS	Post-rTMS	Pre-rTMS	Post-rTMS				
ICARS	41.33 ± 15.45	33.56 ± 15.01	29.33 ± 12.04	27.56 ± 12.43	**<0.001*^e^***	**0.002*^e^***	**0.085*^f^***	**<0.001*^f^***
PG	17.78 ± 6.40	15.78 ± 6.70	12.33 ± 6.63	11.89 ± 6.70	**0.001*^e^***	**0.035*^e^***	**0.095*^f^***	**0.003*^f^***
KF	16.89 ± 7.41	11.22 ± 6.69	12.22 ± 6.04	10.89 ± 6.60	**<0.001*^e^***	**0.004*^e^***	**0.162*^f^***	**<0.001*^f^***
DS	3.89 ± 1.62	3.89 ± 1.62	2.33 ± 1.32	2.33 ± 1.32	/*	**0.041*^e^***	/*	/*
OMS	2.78 ± 0.97	2.67 ± 0.87	2.67 ± 1.32	2.67 ± 1.12	**0.347*^e^***	**1*^e^***	**0.842*^f^***	**0.588*^f^***

ICARS = the International Cooperative Ataxia Rating Scale; PG = posture and gait; KF = limb kinetic function; DS = speech disorders; OMS = oculomotor disorders. Values are expressed as mean ± standard deviation and median (upper limit, lower limit); the value in bold represents statistical significance. ^a^Pre-stimulation vs. post-stimulation in real rTMS. ^b^Pre-stimulation vs. post-stimulation in sham rTMS. ^c^Comparison between groups (pre-stimulation). ^d^Comparison of differences between groups (real vs. sham group). ^e^Paired-samples t test. ^f^Independent samples t test. *No statistical analysis was performed because there was no change in the results before and after.

**TABLE 3:** The NAA/Cr and Cho/Cr values in the real and sham interventions before and after stimulation.

	Real rTMS		Sham rTMS		*P* value^a^	*P* value^b^	*P* value^c^	*P* value^d^
				
Variables	Pre-rTMS	Post-rTMS	Pre-rTMS	Post-rTMS				
**NAA/Cr**								
Ce	0.62 ± 0.16	0.96 ± 0.14	0.64 ± 0.11	0.65 ± 0.10	**<0.001^e^**	**0.560^e^**	**0.842^h^**	**<0.001^h^**
L-DN	0.77(0.64,0.80)	1.03(0.99,1.30)	0.82 ± 0.13	0.81 ± 0.15	**0.018^f^**	**0.412^e^**	**0.197^h^**	**0.018^h^**
R-DN	0.70 ± 0.15	1.15 ± 0.17	0.73 ± 0.18	0.77 ± 0.16	**0.007^e^**	**0.172^e^**	**0.726^h^**	**0.009^h^**
L-CH	0.62 ± 0.11	0.95 ± 0.10	0.69 ± 0.08	0.69 ± 0.06	**<0.001^e^**	**0.914^e^**	**0.154^h^**	**<0.001^h^**
R-CH	0.71(0.67,0.78)	1.10(0.97,1.12)	0.83 ± 0.10	0.82 ± 0.07	**0.018^f^**	**0.658^e^**	**0.027^h^**	**<0.001^h^**
**Cho/Cr**								
Ce	0.81 ± 0.16	1.23 ± 0.17	0.88 ± 0.16	0.84 ± 0.12	**<0.001^e^**	**0.341^e^**	**0.412^h^**	**<0.001^h^**
L-DN	0.92 ± 0.08	1.39 ± 0.39	0.96 ± 0.10	0.97 ± 0.10	**0.021^e^**	**0.899^e^**	**0.325^g^**	**0.021^h^**
R-DN	0.85(0.75,1.00)	1.17(1.11,1.27)	0.89 ± 0.15	0.87 ± 0.13	**0.018^f^**	**0.248^e^**	**0.654^h^**	**0.022^h^**
L-CH	0.86 ± 0.16	1.17 ± 0.08	0.91 ± 0.09	0.89 ± 0.11	**<0.001^e^**	**0.411^e^**	**0.388^h^**	**<0.001^h^**
R-CH	0.88(0.58,0.98)	1.14(1.13,1.23)	0.95 ± 0.12	0.94 ± 0.12	**0.018^f^**	**0.454^e^**	**0.088^h^**	**0.001^h^**

*rTMS = repetitive transcranial magnetic stimulation; Ce = cerebellar vermis; L-CH = left cerebellar hemisphere; R-CH = right cerebellar hemisphere; L-DN = left dentate nucleus; R-DN = right dentate nucleus. Values are expressed as mean ± standard deviation and median (upper limit, lower limit); the value in bold represents statistical significance. ^a^Pre-stimulation vs. post-stimulation in real rTMS. ^b^Pre-stimulation vs. post-stimulation in sham rTMS. ^c^Comparison between groups (pre-stimulation). ^d^Comparison of differences between groups (real vs. sham group). ^e^Paired-samples t test. ^f^Wilcoxon signed rank test. ^g^Mann-Whitney U test. ^h^Independent samples t tests.*

**FIGURE 2:**
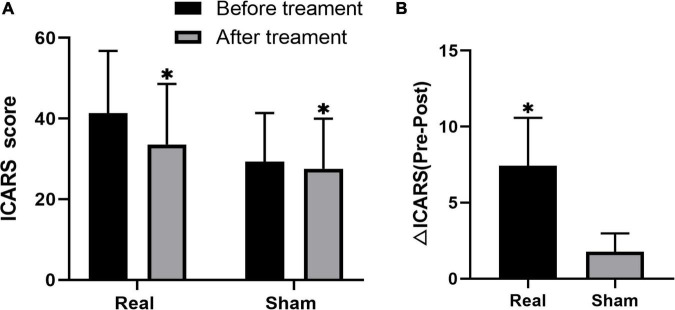
Changes in ICARS score before and after treatment in both groups of real and sham stimulation. The comparison between pre and post stimulation in both groups, respectively **(A)**. The comparison of difference in ICARS (△ICARS) after the stimulation between real and sham stimulation groups **(B)**. *Indicates the significant difference.

The ICARS scores significantly decreased after treatment in both the groups, while the decrease was more significant in the real stimulation group compared to the sham stimulation group (*p* < 0.001) (Figure 2; the comparison of scores of ICARS and its four subscales’ domain before and after stimulation in the real and sham intervention groups are provided in Table 2). To confirm the robustness of the primary findings, we used the analysis of covariance. The total ICARS scores decreased more dramatically in the real group after treatment compared to the sham group (F = 31.239, *p* < 0.001), and three of the four subscales’ domain scores had the same decreased pattern as the total ICARS scores as follows: PG (F = 13.037, *p* = 0.003), KF (F = 22.679, *p* < 0.001), DS (no statistical analysis was performed because there was no change in the results before and after), OMS (F = 0.261, *p* = 0.617).

**FIGURE 3:**
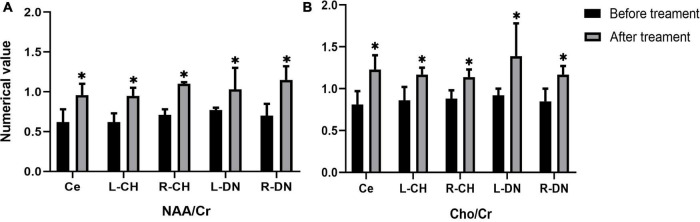
Changes in the values of NAA/Cr **(A)**, and Cho/Cr **(B)** in cerebellar vermis, left and right cerebellar hemisphere, left and right dentate nucleus before and after treatment in the real stimulation group. (Ce representing the cerebellar vermis; L-CH representing the left cerebellar hemisphere; R-CH representing the right cerebellar hemisphere L-DN represents the left dentate nucleus; R-DN represents the right dentate nucleus). *Indicates the significant difference in the comparison between pre and post stimulation.

**FIGURE 4:**
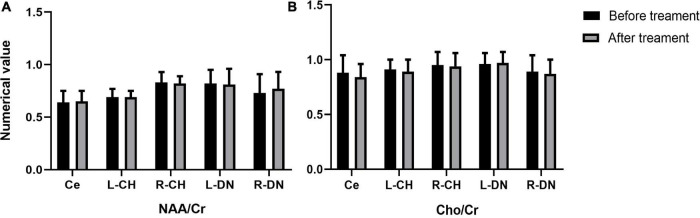
Changes in the values of NAA/Cr **(A)**, and Cho/Cr **(B)** in cerebellar vermis, left and right cerebellar hemisphere, left and right dentate nucleus before and after treatment in the sham stimulation group (Ce representing the cerebellar vermis; L-CH representing the left cerebellar hemisphere; R-CH representing the right cerebellar hemisphere L-DN represents the left dentate nucleus; R-DN represents the right dentate nucleus).

In the real stimulation group, after 15 consecutive days of low-frequency rTMS treatment, the NAA/Cr values in the cerebellar vermis, left and right lateral in the cerebellar hemispheres, and dentate nuclei were all significantly elevated, compared with those before treatment (*p* < 0.05) (Table 3 and Figure 3A). The Cho/Cr values in the above-mentioned brain regions were also obviously elevated compared to those before treatment (*p* < 0.05) (Table 3 and Figure 3B); on the other hand, in the sham stimulation group, there were no significant differences in either the NAA/Cr and Cho/Cr values in the brain regions noted above both before and after stimulation (Table 3 and Figure 4). The elevations in the NAA/Cr and Cho/Cr values after the stimulation were significantly higher in the real stimulation group than in the sham stimulation group (Table 3). The mean change in MRS after treatment using covariance (ANCOVA) showed that the NAA/Cr and Cho/Cr values increased after treatment in the real group compared to the respective scores in the sham group (Ce-NAA/Cr: F = 72.4, *p* < 0.001; Ce-Cho/Cr: F = 73.70, *p* < 0.001; L-CH-NAA/Cr: F = 10.38, *p* = 0.007; L-CH-Cho/Cr: F = 9.41, *p* = 0.009; R-CH-NAA/Cr: F = 22.6, *p* < 0.001; R-CH-Cho/Cr: F = 16.14, *p* = 0.001; L-DN-NAA/Cr: F = 48.24, *p* < 0.001; L-DN-Cho/Cr: F = 61.41, *p* < 0.001; R-DN-NAA/Cr: F = 25.2, *p* < 0.001; R-DN-Cho/Cr: F = 61.15, *p* < 0.001) (Refer to Table 3 for abbreviations).

In the real stimulation group, the NAA/Cr value changes in the right cerebellar hemisphere were negatively correlated with the changes in the ICARS scores (r = − 0.831, *p* = 0.02), while the changes in the NAA/Cr values in the left cerebellar hemisphere did not correlate with the changes in the ICARS scores (r = − 0.495, *p* = 0.259). No other correlation existed between the remaining indicators.

## Discussion

In this study, we conducted a randomized, double-blind, controlled trial among SCA3 patients and found that after the low-frequency rTMS intervention, the NAA/Cr and Cho/Cr values increased and the ataxia symptoms improved. In addition, there was a correlation between the changes in the NAA/Cr values and ICARS scores. Investigating the changes of cerebellar neurochemicals in the SCA3 patients under rTMS intervention is an innovation developed in recent years.

In clinical practice, MRS can non-invasively detect changes in metabolite concentrations in the brain tissue and accurately detect NAA, Cho, and Cr in the brain, providing reliable biomarkers for SCA3 (Matos et al., 2019). The NAA represents neuronal viability and integrity (Wang et al., 2012), and creatine (Cr) reflects energy metabolism in the brain (Wang et al., 2012); Cr concentrations in the brain tissue are high and relatively stable. Therefore, NAA and Cr can be used as a reference for comparison between the groups. Choline (Cho) is a marker of cell renewal (Bridges et al., 2018). NAA/Cr indicates neuronal function at the test site, and Cho/Cr reflects glial cell function; therefore, NAA/Cr and Cho/Cr can be used as indicators for neuronal and myelin integrity (Cuypers and Marsman, 2021). Significant pathological damage and atrophy in the patients with SCAs have been shown to result in neuronal loss/dysfunction, and therefore, a decrease in the NAA and Glu (Adanyeguh et al., 2015).

There are studies on the changes in the cerebellar neurometabolites in some diseases under low-frequency rTMS intervention. However, there were no published studies on the changes in the cerebellar neurometabolites in the SCA3 patients with low-frequency rTMS intervention. For the treatment of cognitive function associated with cranial injury, the patient’s MRS showed a decrease in both NAA/Cr and Cho/Cr ratios (Zhou et al., 2021). Low-frequency rTMS interfered with *N*-acetyl aspartate (NAAG) synthesis and cell turnover (Bridges et al., 2018) as well as increased NAA levels in the prefrontal and striatal lobes (Hone-Blanchet et al., 2017). Low-frequency rTMS leads to selective changes in the glutamate and gamma-aminobutyric acid concentrations in different brain regions (Yue et al., 2009). It may improve the metabolic activity of NAA and other neuronal cells by stimulating the cortical neurons.

The mechanism by which rTMS affects the neural metabolism in the brain of SCA patients can be explained as follows: low-frequency rTMS inhibits cortical excitability. However, a previous study showed that 1 Hz rTMS led to an increase in the NAA rather than the expected decrease, which may be due to an *in vivo* homeostatic mechanism wherein inhibitory 1 Hz rTMS may paradoxically induce an increase instead of a decrease in the local activity (Fregni et al., 2011). Research has shown that low-frequency rTMS increased brain-derived neurotrophic factor content and nerve growth factor expression, resulting in increased neurometabolic substances (Tan et al., 2013). Among them, the increase in the tCho/tCr ratio may be related to underlying neuroplasticity processes (Flamez et al., 2019). Studies have shown that rTMS can improve motor function in patients, likely because continuous stimulation with rTMS at the same frequency and intensity can cause uninterrupted excitation or inhibition of neuronal cells, allowing neural networks to reorganize after brain injury, and thus improving clinical symptoms (Zhou et al., 2021). From this, we can speculate that the possible mechanism of low-frequency rTMS in treating SCA3 patients should improve neuronal cell function by modulating the ability of gene expression and regulating the expression of various growth factors.

The Scale for the Assessment and Rating of Ataxia (SARA) is currently the tool of choice to demonstrate the efficacy of the SCA3 disease improvement or symptomatic treatment for ataxia, the most important disease feature, and numerous clinical studies have identified SARA as the preferred method (Saute and Jardim, 2018; Lin et al., 2019; Diallo et al., 2021; Maas et al., 2021). It has also been shown that ICARS and SARA are reliable and valid scales for assessing the severity of ataxia in patients with SCA3/MJD and that the most appropriate scale can be selected according to specific requirements (Zhou et al., 2011). ICARS shows very high inter-rater reliability and is sensitive to a wide range of ataxia symptoms, from very mild to severe (Zhou et al., 2011). There is a correlation between it and the degree of cerebellar lesions (Xing et al., 2017). ICARS outperforms SARA in terms of responsiveness in SCA patients (Perez-Lloret et al., 2021), and many interventional studies have used ICARS alone (Peng et al., 2019; de Oliveira et al., 2021; Leotti et al., 2021), thus, we ultimately chose to use ICARS as the primary clinical outcome parameter.

Both the real and sham groups demonstrated improvement in the ICARS scores. There are many possible reasons behind the improvement in the sham group. First, there may be a placebo effect, which is very common in the intervention studies of cerebellar degeneration (Bier et al., 2003; Zesiewicz et al., 2012); the sham stimulation produced the same noise as the real stimulation and had some scalp perception. Patients were unaware of the difference between the real and sham stimulation because no patient had previously experienced real stimulation. Therefore, patients undergoing sham stimulation did not notice that they were receiving inactive stimulation. Second, all patients were under the supervision of clinical research staff during the study period, which may have been more than their normal care. This may have induced underlying psychological factors in the patients that could have produced a significant treatment effect (Manor et al., 2019). Third, repeated assessment of ICARS over a short time may lead to patient proficiency in the assessment methods, which may then produce improved effects beyond stimulation. We also found an interaction between time and intervention in our results, which suggests a difference in the trend of the outcomes ICARS over time in the two groups. The effect of true stimulation may become more significant as the duration of the intervention increases, but we measured only one time point after treatment, so there may be bias.

Nevertheless, this study has several limitations. First, given that the sample size was small, the evidence collected was limited. Second, since the participants did not receive follow-up care after the study, we were not able to measure the long-term efficacy of low-frequency rTMS on the cerebellar metabolites. Third, although the participants were blind to the assigned treatment, we used a true coil in the sham stimulation group, placed differently than in the real stimulation group, and the patients might have become aware of this difference during the study. This limitation needs to be considered regarding the generalization of the study results. Finally, we conducted a double-blind trial, but the therapist doing the rTMS was not blinded, which may affect the objectivity of the study.

## Conclusion

Overall, the treatment with rTMS over the cerebellum induced changes in cerebellar local metabolism and microenvironment in SCA3 patients. The alterations may contribute to the improvement of the ataxia symptoms in SCA3 patients.

## Data Availability Statement

The raw data supporting the conclusions of this article will be made available by the authors, without undue reservation.

## Ethics Statement

The studies involving human participants were reviewed and approved by the Ethics Committee of First Affiliated Hospital of Fujian Medical University (MRCTA, ECFAH of [2018]201). The patients/participants provided their written informed consent to participate in this study.

## Author Contributions

X-YC, Y-HL, and X-HL for designed the study, wrote the manuscript, and processed the data. AS and H-LX helped in the transcranial magnetic intervention and patient assessment. J-PH and M-CL contributed to the magnetic resonance scanning and processing. Q-LC and S-RG helped in manuscript preparation and contributed to the supervision of the whole process. All authors reported above for publication was completed and contributed to the article and approved the submitted version.

## Conflict of Interest

The authors declare that the research was conducted in the absence of any commercial or financial relationships that could be construed as a potential conflict of interest.

## Publisher’s Note

All claims expressed in this article are solely those of the authors and do not necessarily represent those of their affiliated organizations, or those of the publisher, the editors and the reviewers. Any product that may be evaluated in this article, or claim that may be made by its manufacturer, is not guaranteed or endorsed by the publisher.
